# Use of Laparoscopic and Laparotomic J-Plasma Handpiece in Gynecological Malignancies: Results From A Pilot Study in A Tertiary Care Center

**DOI:** 10.3389/fonc.2022.868930

**Published:** 2022-06-28

**Authors:** Salvatore Gueli Alletti, Andrea Rosati, Vito Andrea Capozzi, Matteo Pavone, Alessandro Gioè, Stefano Cianci, Vito Chiantera, Giuseppe Vizzielli, Giulia Scaglione, Anna Fagotti, Giovanni Scambia

**Affiliations:** ^1^Dipartimento per la salute della Donna e del Bambino e della Salute Pubblica, Fondazione Policlinico Universitario A. Gemelli, IRCCS, UOC Ginecologia Oncologica, Rome, Italy; ^2^Università Cattolica del Sacro Cuore, Rome, Italy; ^3^UOC Ginecologica e Ostetricia, Dipartimento Materno-Infantile, Ospedale Buccheri La Ferla Fatebenefratelli, Palermo, Italy; ^4^Department of Gynecology and Obstetrics of Parma, University of Parma, Parma, Italy; ^5^Department of Gynecologic Oncology and Minimally-Invasive Gynecologic Surgery, Università degli studi di Messina, Policlinico G. Martino, Messina, Italy; ^6^Department of Gynecologic Oncology, Aziende di Rilievo Nazionale di Alta Specializzazione, Civico Di Cristina Benfratelli, Palermo, Italy; ^7^Department of Gynecologic Oncology, Università di Palermo, Palermo, Italy; ^8^Obstetrics, Gynecology and Pediatrics Department, Udine University Hospital, DAME, Udine, Italy; ^9^Department of Woman, Child and Public Health Sciences, Gynecopathology and Breast Pathology Unit, Fondazione Policlinico Universitario A. Gemelli IRCCS, Rome, Italy

**Keywords:** J-Plasma, argon, cytoreductive surgery, laparoscopic surgery, ovarian cancer, endometrial cancer, laparoscopy, laparotomy

## Abstract

**Introduction:**

The J-Plasma has recently been introduced into the surgical community with different intrinsic characteristics aimed to further reduce the thermal effect and enhance precision when compared to standard radiofrequency. This study aimed to investigate the role of this new technology in different conditions of gynecological carcinomatosis characterized by the indication for regional peritonectomy and/or ablation, either in laparotomy (LPT) or in laparoscopy (LPS), in the context of a modern personalized approach to the surgical management of gynecological malignancies.

**Material and Methods:**

From January 2019 to April 2019, 12 patients were selected for this prospective pilot study at the Division of Gynecologic Oncology, Fondazione Policlinico Universitario A. Gemelli IRCCS in Rome. In this single surgeon experience, the inclusion criteria were: histologically proven advanced ovarian/endometrial cancer, primary or interval debulking surgery, and intraoperative indication for regional peritonectomy. Six patients were treated by LPS (Group 1) and 6 by LPT (Group 2).

**Results:**

In Group 1 the indication for debulking surgery was in 4 cases an interval debulking surgery and 2 advanced endometrial cancer. All patients in Group 2 underwent primary debulking surgery for advanced ovarian cancer. The whole cohort achieved a complete tumor excision after surgery. The median OT and median EBL were 195 min and 100 ml in Group 1, and 420 min and 500 ml in Group 2. The median hospital stay was 4 days in Group 1 and 13 days in Group 2, respectively. No intra and postoperative complications were registered within 60 days after surgery.

**Conclusions:**

J-Plasma allows to approach delicate maneuvers on viscera, mesentery, and blood vessels with a high degree of safety and precision thanks to its limited vertical and lateral thermal spread, favoring the surgeon to push ever higher the cytoreduction/morbidity tradeoff. The use of J-Plasma in cytoreductive surgery could also increase the range of possible minimally invasive procedures, narrowing the technical distance with the open technique and thus contributing to designing a personalized surgical strategy for each patient in different scenarios of peritoneal carcinomatosis.

## Introduction

In the last decade, constant technological progress has continuously supported gynecological surgeons in optimizing their surgical performance and pushing the effectiveness of the treatment of gynecological malignancies higher. Even if, to date, both laparoscopy (LPS) and laparotomy (LPT) have specific indications for treating gynecological malignancies, their role is in constant evolution (1–3). In recent years, many published experiences have demonstrated that the proper instruments in the hands of an experienced gynecological oncosurgeon can reduce the distance between both approaches with superimposable results in terms of perioperative and oncological outcomes (4).

In the case of specifically designed trials, the laparoscopic approach was found to be advantageous compared with standard laparotomy (5, 6). The surgical treatment of peritoneal carcinomatosis, both from ovarian and endometrial cancer, remains the most demanding factor, limiting the surgical options for the surgeon and patient. When regional peritoneal carcinomatosis is observed, the proper instrumentation, with the surgical experience, can provide the opportunity to shift from extensive median laparotomy to a minimally invasive setting with maintained oncological adequacy. Given this, LPS and LPT need to be considered as different options within an integrated, personalized treatment. In each case, the choice of the right instrumentation to achieve the surgical purpose is one of the most demanding factors influencing the surgical performance and, thus, the outcome of the patient. The classical monopolar electrosurgery, widely used for peritoneal resection and retroperitoneal dissections, is often inappropriate because of several issues: the lateral and depth of thermal spread on the bowel surface; the carbonization with subsequent inflammation in extended peritonectomies; and the galvanic effect on muscle surfaces (7, 8). These characteristics limit its use in the most vulnerable anatomical areas. In this surgical context, the use of argon plasma has been classically associated with a specific condition requiring extensive peritonectomy or tissue ablation in laparotomic cytoreduction (9, 10).

However, its use has been limited due to the extensive lateral thermal spread with the risk of injuries to adjacent tissues (11). The J-Plasma (Apyx Medical Corp.) has been recently introduced into the portfolio of the surgical community with different intrinsic characteristics, further reducing the thermal effect by delivering a focused helium stream energized by radiofrequency. This novel platform provides modified plasma energy with a tissue effect different from the classical argon plasma. The cool atmospheric plasma stream is generated by the helium gas passing across an energized electrode to create a focused stream. The contactless energy application, with low voltages and low gas flow, ensures a minimal lateral thermal effect depending on the duration of the application (12, 13). In addition, the presence of a retractable tip allows the targeting of the surgical site, further increasing the precision of energy delivery. The availability of different handpieces for both open and LPS surgery enables thin-layer ablation, dissection, and coagulation in different surgical situations. The application of J-Plasma in gynecologic oncology is limited to preliminary experiences regarding its effective and safe use in performing diaphragmatic peritonectomy during upper abdominal procedures for advanced ovarian cancer (14).

Therefore, we designed a pilot study deeply investigating the role of this new technology in different conditions of gynecological carcinomatosis characterized by the indication for regional peritonectomy, either in laparotomy or in laparoscopy, in the context of a modern personalized approach delivered at a tertiary care referral center.

## Materials and Methods

This pilot study was conducted at the Division of Gynecologic Oncology, Fondazione Policlinico Universitario A. Gemelli IRCCS in Rome, between January 2019 and April 2019. During the study period, 12 patients were prospectively enrolled in the study (15). The Institutional Review Board has approved the study (no. DIPUSVSP-03-11-2184). The inclusion criteria were histologically proven advanced ovarian/endometrial cancer, primary or interval debulking surgery, and intraoperative indication for regional peritonectomy.

Written informed consent was obtained from all individual participants before the procedures and permission for the publication was also taken in accordance with the 1964 Helsinki Declaration and its later amendments or comparable ethical standards. All surgical procedures were performed with the assistance of the J-plasma handpiece, either laparoscopic or laparotomic, when indicated. The surgery was performed, both in LPS and LPT, by a single high-volume surgeon (more than 50 procedures per year) (16). The standard power setting of the device was Coagulation: 45, Cut: 45, Bipolar Macro: 1, J-Plasma Power: 40%, Gas Flow: 4.0 l/min, and Pulse: 80. J-plasma was applied in a different surgical situation where a thin layer of dissection, coagulation, or vaporization was needed (**Video 1**). We collected two peritoneal biopsies. All surgical specimens were sent to the Histopathology and Cytodiagnostic Unit, where processed tissue was formalin-fixed, paraffin-embedded (FFPE), and finally stained with hematoxylin and eosin (H&E). Baseline and perioperative variables were prospectively collected for each patient. Early post-operative complications (arising within 30 days of surgery) were classified using the extended Clavien–Dindo classification of surgical complications (17). Descriptive statistics were used to describe the clinical, demographic, and surgical variables. Qualitative variables have been summarized as absolute and percentage frequencies; quantitative variables have been summarized by their median and range.

## Results

Twelve patients were enrolled during the study period. Among them, six were treated by laparoscopy (Group 1) and six by laparotomy (Group 2). **Table 1** shows the baseline characteristics of the study population. The median age was 67 and 58, respectively, in Group 1 and Group 2, while the median BMI was 24 kg/m^2^ in the LPS group and 30 kg/m^2^ in the laparotomic group. In Group 1, four cases were interval debulking surgery (IDS), and two had advanced endometrial cancer with pelvic carcinomatosis (Stage IV). Conversely, all patients in Group 2 underwent laparotomic primary debulking surgery for advanced-stage ovarian cancer.

**Table 1:** Patients’ baseline characteristics and indications for surgery.

	Group 1 (LPS)n = 6	Group 2 (LPT) n = 6
Number of Patients	6	6
Age median (range)	67 (64–77)	58 (54–68)
BMI median (range)	24 (22–31)	30 (27–31)
**Indications for surgery**		
PDS in AEOC	–	6
IDS in AEOC	4	–
Stage IV EC	2	–

Regarding the surgical outcomes (**Table 2**), we reported a median operative time of 195 and 420 min, and a median EBL of 100 and 500 ml, respectively, in Group 1 and Group 2. The different surgical indications and the different settings in which the two surgical approaches occurred determined a clustering of the more complex procedures such as colo-rectal resections, splenectomy, distal pancreatectomy, and lesser omentum excision in Group 2. However, in Group 1, we also reported two cases of diaphragmatic peritonectomy during a laparoscopic interval debulking surgery in which the affected peritoneum was localized in the ventral portion of the diaphragmatic dome. The device was used in both groups in specific surgical steps requiring high precision to avoid iatrogenic damage to noble structures. More in detail, J-Plasma was used during parietal/pelvic peritonectomy (in all cases of Group 1 and 4 patients in Group 2), diaphragmatic peritonectomy (two in Group 1 and in all cases of Group 2), mesenteric peritonectomy (two cases in both groups), the ablation of nodules on the bowel serosa (two cases in the laparotomic group), and twice to perform laparoscopic pelvic lymphadenectomy **(** **Video 1**; **Supplementary Material****).** The goal of complete cytoreduction to no gross residual disease was reached in the whole study population. The median hospital stay was 4 days for Group 1 and 13 days for Group 2, respectively. No intra- or postoperative complications were registered within 60 days after surgery.

**Table 2:** Perioperative variables and J-Plasma assisted surgical procedures.

	Group 1 (LPS) n = 6	Group 2 (LPT) n = 6
Operative time (min) Median (range)	195 (180–210)	420 (360–480)
EBL (ml) Median (range)	100 (50–200)	500 (400–1,200)
Complete cytoreduction (RT = 0)	6	6
Hospital stay (day) Median (range)	4 (4–5)	13 (7–16)
**Overall Surgical procedures**		
Radical omentectomy	4	4
RH + BSO	6	6
Pelvic Lymphadenectomy	2#	4
Colo-rectal resection	0	4
At least one complex surgical procedure in the upper abdomen*	0	4
**Surgical procedures with J-Plasma**		
Parietal/pelvic peritonectomy	6	4
Diaphragmatic peritonectomy	2	6
Mesenteric peritonectomy	2	2
Bowel serosa ablation	–	2

*Splenectomy, distal pancreasectomy, lesser omentum excision.

#Performed with J-Plasma.

## Discussion

In this study, we show how the novel technology of cool plasma can be applied to different surgical conditions characterized by diffused or regional carcinomatosis. Indeed, all patients showed peritoneal/serosal carcinomatosis with the indication both for extensive or regional peritonectomy and/or ablation with the assistance of J-Plasma. Ten of twelve cases of peritoneal carcinomatosis were advanced ovarian cancers; the other two cases showed pelvic carcinomatosis for endometrial cancer.

In this context, ovarian cancer represents the most challenging disease in terms of strategical management, surgical invasiveness, oncological adequacy, and integrated treatment. If LPT is the standard approach for cytoreductive surgery in gynecological carcinomatosis, minimally invasive surgery (MIS) plays a specific and emerging role in each surgical step along with the natural history of these conditions (1–6). Moreover, the international pioneer experiences have recently got a breach to MIS approach in selected cases of IDS (4). Nevertheless, the role of the minimally invasive approach in IDS is a hotly debated and ongoing topic. The advantage of laparoscopy lies in the reduction of incisional pain and a faster recovery. However, it is necessary to consider numerous variables to modulate its proper application, such as the presence of diffuse peritoneal adhesions, the anatomical location and number of disease nodules, the experience of the surgeon, and the biochemical and radiological response to NACT (18, 19).

In this scenario, the surgeon must constantly perform many complex surgical maneuvers during laparoscopy that could benefit from advanced technical support. We must also consider that the transcoelomic metastatic pathway usually results in the spread of carcinomatosis on the diaphragmatic surface (40% of patients with advanced disease), mesentery root, small/large intestine, and parietal peritoneum (20). Diffuse involvement of those structures is considered one of the most demanding hurdles to overcome to achieve a complete cytoreduction. Traditionally, the stripping or cauterization of the affected peritoneum is performed by conventional monopolar electrocautery with several limitations and risks. Often, the contact of tissue with the metal surface of the device results in the adhesion of the charred tissue to the device itself. The subsequent removal of the metal can snatch away the charred tissue, causing a re-bleed (21). In addition, the current produced by conventional monopolar electrocautery has an uneven tissue distribution at sites of entry and can cause unexpected oxidation/charring of adjacent tissues. An unpredictable depth of injury is unacceptable at vulnerable anatomical sites such as the bowel wall, mesentery, ureters, major blood vessels, and diaphragm. Therefore, new technologies have been devised to offer a more superficial and controlled type of energy transmission to assist the surgeon in precise plane dissection. The first step was represented by the Argon beam coagulator (monopolar current *via* a beam of inert argon gas). It was introduced in AOC cytoreductive surgery in the 1990s with the purpose of delivering energy in a more homogeneous and predictable fashion compared to classic electrosurgical devices (22).

Although many studies deeply investigated its role in achieving complete cytoreduction and reducing morbidities (23, 24), it was gradually confined to a niche application in gynecologic oncology. The introduction of J-Plasma renewed interest in this type of supply. This is partially due to the evolved approach to carcinomatosis in view of the emerging evidence concerning surgery after neoadjuvant chemotherapy (25). Compared to the Argon beam coagulator, the cold plasma technology shows an even more predictable tissue effect (in terms of amplitude and depth of coagulation), less inflammatory response, no histological evidence of perivascular hemorrhages or focal damage to the blood circulation while leaving surrounding tissues intact (13). Another crucial point is the possibility to customize the power and flow of the stream accordingly to the characteristics and extent of carcinomatosis. The benefits of the precise cutting and minimal thermal spread are reflected in the possibility of surgeons to more aggressively treat lesions close to vulnerable anatomical structures, raising the rate of complete cytoreduction without increasing the perioperative morbidity and mortality (26). Moreover, based on our results, we can also infer that cold plasma energy could allow the surgeon to extend the range of surgical maneuvers performed using a minimally invasive approach. Accordingly, with our results, the use of plasma energy facilitates the removal of disease in the bowel serosa (rectal shaving), intestinal mesentery, diaphragmatic region, and during pelvic lymphadenectomy both in laparoscopic and laparotomic approaches (27, 28).

In this single experience of a surgeon, the learning curve was reached within three procedures owing to the ergonomic and intuitive shape of the device: we assume that superimposable results can be achieved in wider experiences performed by high-volume gynecological oncosurgeons.

**Figure 1:**
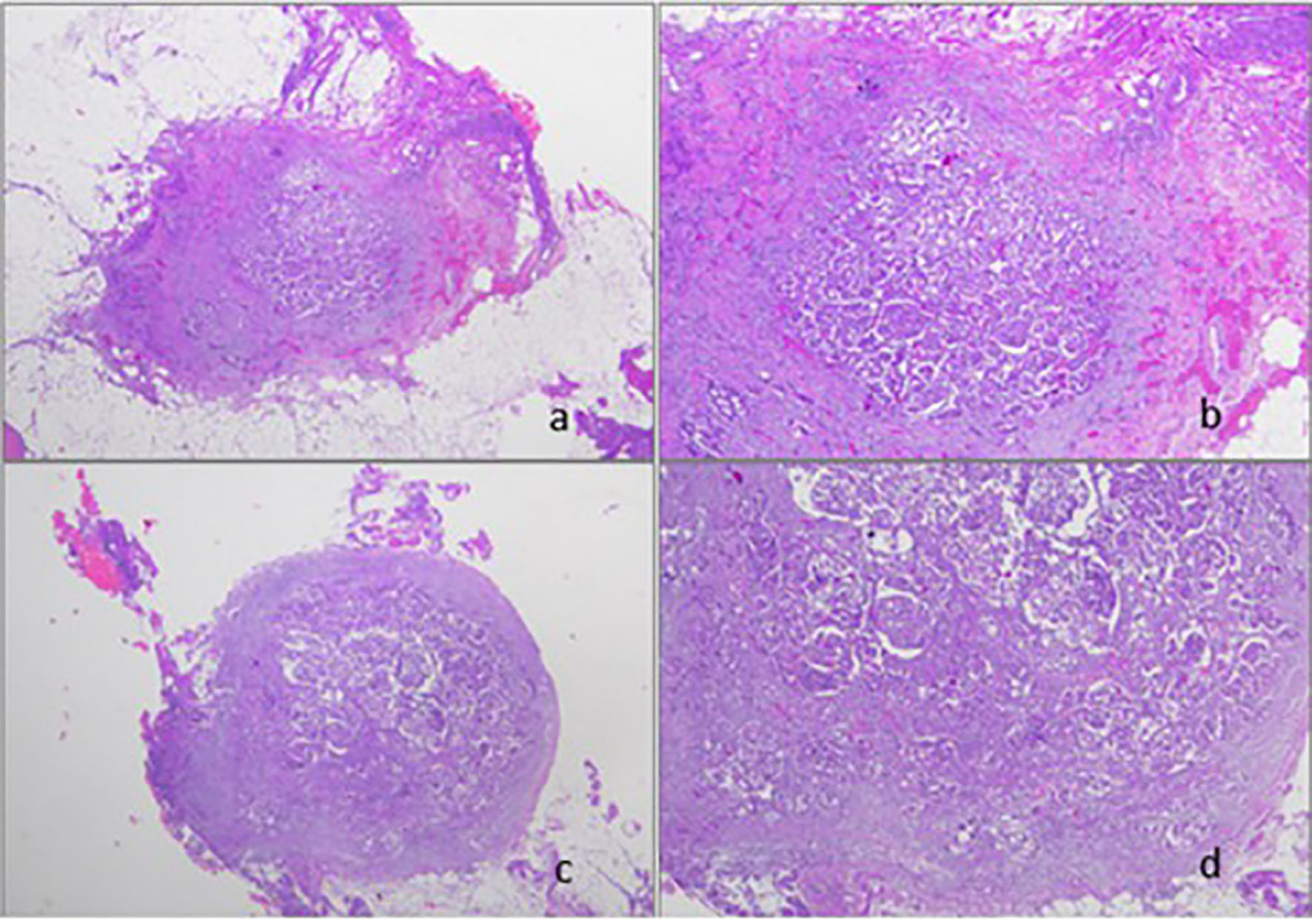
**(A, B)** Peritoneal biopsy performed by standard electrosurgery stained by hematoxylin–eosin (original magnification ^x^50 and ^x^200). **(C, D)** Peritoneal biopsy performed by J–plasma surgery stained by hematoxylin-eosin (original magnification ^x^50 and ^x^200).

To more broadly discuss the oncological concern, the “non-thermal effects” of plasma (or cold plasma effect), such as plasma-induced apoptosis and the decrease in cell migration velocity, could have important implications in cancer treatment by destroying the affected area while decreasing metastatic development (29,30). In **Figures 1A, B**, peritoneal biopsy, hematoxylin–eosin stained, shows a post-neoadjuvant chemotherapy serous ovarian carcinoma. On histological slides, irregular resection margins and extensive tissue damage due to electrosurgery were noted.

Peritoneal biopsy performed by the J-Plasma surgical device (**Figures 1C, D**) histologically showed cleaner edges and less tissue damage than conventional electrosurgery specimens. Besides, we observed residual tumor after chemotherapy with nested-solid growth and marked atypia.

The main strength of our study is represented by the prospective nature and the innovative subject in the field of technological advancement, while limitations lie in the limited number of patients and the presence of two different types of gynecological tumors treated.

## Conclusions

Controlling energy and applying it adaptively to different tissues allows technological advancement to promote the personalization of surgical treatment. Modulating the energy source, such as monopolar, bipolar, integrated, or plasma, to a specific task in the context of a “fluid” surgical strategy is the struggle of the surgeon to push ever higher the cytoreduction/morbidity tradeoff. In this context, J-Plasma allows approaching viscera, mesentery, and blood vessels with high safety and precision thanks to a limited vertical penetration into the tissue and a minimal lateral thermal effect. Moreover, the tunable tissue impact of the energized helium flow also greatly increases the safety profile, allowing its activation close to noble vascular structures. Even if the small number of patients included is a limitation of this pilot study, we show how J-Plasma seems advantageous during specific cytoreductive maneuvers. It can also theoretically increase the range of possible MIS procedures by narrowing the technical distance with the open technique. Nonetheless, further experience and prospective studies must clarify the full potential of J-Plasma in cytoreductive surgery for gynecological cancers.

## Data Availability Statement

The raw data supporting the conclusions of this article will be made available by the authors, without undue reservation.

## Ethics Statement

The studies involving human participants were reviewed and approved by the Institutional Review Board approbation no. DIPUSVSP-03-11-2184. The patients/participants provided their written informed consent to participate in this study.

## Author Contributions

SGA is responsible for study design, drafting of the manuscript, and data interpretation. AR is responsible for study design, drafting of the manuscript, and data interpretation. VC is responsible for the acquisition and quality control of data MP is responsible for study design and the acquisition and quality control of data. CS is responsible for the acquisition and quality control of data. CV is responsible for the critical revision of the manuscript for important intellectual content. GV is responsible for the critical revision of the manuscript for important intellectual content. GS is responsible for the acquisition and quality control of data. GS is responsible for the critical revision of the manuscript for important intellectual content. AF is responsible for the critical revision of the manuscript for important intellectual content. All authors listed have made a substantial, direct, and intellectual contribution to the work and approved it for publication.

## Conflict of Interest

The authors declare that the research was conducted in the absence of any commercial or financial relationships that could be construed as a potential conflict of interest.

## Publisher’s Note

All claims expressed in this article are solely those of the authors and do not necessarily represent those of their affiliated organizations, or those of the publisher, the editors and the reviewers. Any product that may be evaluated in this article, or claim that may be made by its manufacturer, is not guaranteed or endorsed by the publisher.
